# Dark Archives: A Librarian's Investigation into the Sci-ence and History of Books Bound in Hu-man Skin

**DOI:** 10.5195/jmla.2022.1563

**Published:** 2022-07-01

**Authors:** Megan Nance

**Affiliations:** 1 megan.nance@yale.edu, Cushing/Whitney Medical Library, Yale University, New Haven, CT.

For most, the ghoulish concept of anthropodermic bibliopegy, or the practice of binding books in human skin, often conjures fictional imagery of a witch's grimoire or H.P. Lovecraft's famous Necronomicon. However, to assume that *Dark Archives: A Librarian's Investigation into the Science and History of Books Bound in Human Skin* will appeal only to fans of the macabre would be an underestimation of the content covered therein. Throughout the book, the reader follows author Megan Rosenbloom, a journalist and UCLA librarian, as her research takes her to libraries and cultural institutions within the United States, England, and France where she engages with librarians, historians, and leather craftsmen to uncover the science and history of these grisly artifacts.

The book begins by providing readers with an understanding of the leathermaking process as well as the science which has been used to determine the authenticity of human skin texts and the subsequent inception of the Anthropodermic Book Project. Daniel Kirby, a chemist with former ties to the pharmaceutical world, developed an inexpensive and highly precise enzyme testing technique that can be utilized to analyze the collagen proteins found within a leather sample to accurately identify species. Utilizing this method, Kirby worked to authenticate the first scientifically confirmed example of anthropodermic bibliopegy and shortly thereafter, collaborated with Rosenbloom to establish the Anthropodermic Book Project, an initiative that aims to identify and authenticate human skin texts.

From this point, the book swiftly segues into the highly debated question of the preservation and stewardship of these items which often falls within one of two very salient categories: should these texts, which were created using materials that were taken without consent, be preserved, or finally laid to rest? Some librarians, such as Paul Needham of Princeton University, find the books to be abhorrent examples of the predatory medical practices of the nineteenth century and feel as though the texts should be rebound and the human remains buried. Others find the historical significance inherent in these artifacts too revealing to destroy. The author's research makes a convincing argument for the preservation of these pieces as her investigation has uncovered a significant amount of valuable historical contexts relating to the clinical gaze, Victorian medicine, medical consent, and more.

Perhaps most alarming, is the elucidation that the individuals creating these morbid manuscripts were not monsters or psychopaths but trusted, and often highly regarded, doctors and physicians. A detail that shines an uncomfortable light on the early history of medical practice and the distinctly sterilized clinical gaze of Victorian physicians. Rosenbloom works to posture the Victorian-era medical practice accurately as a rising professional class in which many practitioners were eager to prove themselves as respectable, knowledgeable, and capable. The emphasis placed on establishing oneself among society's upper echelon is responsible for several questionable clinical behaviors including the early medical profession's employment of grave robbers and body snatchers to supply corpses for dissection as well as the removal of a patient's skin for the purposes of bookbinding.

Structured in vignettes, Rosen-bloom works to rehumanize these texts by systematically working backward through time to reunite these objects with their names and individual histories. Each essay works to identify the individual whose skin was used to cover the text in question and provide the reader with an understanding of who the individual was and how they became unwilling participants in one of both bookbinding's and medicine's darkest chapters. Rare books and book collecting were central characteristics of scholars, doctors, and gentlemen alike. As such, doctors would occasionally use the skin of ill-fated patients to cover their own medical writings to simultaneously further both their scholarly and rare book pursuits, firmly placing them amongst society's gentleman class. Multiple factors determined a patient's candidacy for medical dissection including their sex, race, or socioeconomic standing—as a result; the skins used to cover texts were most often taken from women, criminals, and the poor.

Readers are first introduced to Mary Lynch, a young Irish woman from a poor family, who was the first known case of trichinosis. Following her death in 1869, Dr. John Stockton Hough, a specialist in women's health, removed and preserved a portion of Lynch's skin which he later used to cover three books relating to the topic of women's health. Readers are then introduced to a host of individuals including Phillis Wheatly, an emancipated enslaved woman and one of the first female African American authors, whose book of poetry is the only title confirmed to have multiple copies bound in human skin. Rosen-bloom is careful to provide space for victims of all circumstances and backgrounds including murderers William Corder and William Burke whose bodies were dissected and distributed as murderabilia and a career criminal, George Walton, the only individual who requested that his skin be used for bookbinding.

*Dark Archives* draws a bold line connecting the absence of empathy in the early medical profession and medical exploitation while also calling attention to what can happen when medical practitioners value their practice over their patients. Rosenbloom's empathy, affable tone, and likable self-awareness helps to present her research in a surprisingly approachable and accessible fashion. Throughout the book, Rosen-bloom works to transition the readers interest from the shocking objects themselves to the people and circumstances that created them. Most notably, Rosenbloom works to provide context, not only, relating to the history, methodology, and significance of these gruesome texts but also raises questions regarding the ethics, consent, and legality of human skin texts while simultaneously challenging our perception of death.

